# Quality of Work Life of Medical Residents: Evaluation of Positive Learning and Working Environment in a Tertiary Hospital

**DOI:** 10.7759/cureus.106981

**Published:** 2026-04-13

**Authors:** Jessica Estefania Silva-Guzman, Lino Palacios-Cruz, Rodolfo Rivas-Ruiz, Eduardo Robles-Pérez, Victor Hugo Borja-Aburto

**Affiliations:** 1 Department of Medical Education and Research, American British Cowdray Medical Center, Mexico City, MEX; 2 Department of Clinical Epidemiology, National Institute of Psychiatry “Ramón de la Fuente Muñiz”, Mexico City, MEX; 3 Health Research Coordination, Mexican Social Security Institute, Mexico City, MEX; 4 Faculty of Medicine, National Autonomous University of Mexico, Mexico City, MEX; 5 Epidemiology and Public Health, Federal Commission for Protection Against Health Risks, Mexico City, MEX

**Keywords:** clinical learning environment, cross-sectional study, medical residency, quality of work life, resident well-being, working environment

## Abstract

Background

Quality of work life (QoWL) is a construct assessing the relationship between working conditions and employee well-being. Resident physicians are a vulnerable group due to high-risk hospital environments and demanding academic-labor dualities. This study is based on the premise that “it is impossible to prevent or optimize what has not been measured.” This study aimed to characterize and identify the environmental, organizational, and personal factors associated with a satisfactory QoWL among resident physicians in a tertiary care hospital.

Methodology

An analytical cross-sectional study was conducted among 210 resident physicians. QoWL was measured using the validated CVT-GOHISALO instrument, which evaluates seven multidimensional domains through 74 items. The primary outcome, “satisfactory QoWL,” was defined as a McCall T-score ≥60. Additional data on clinical and occupational history were collected via the Epidemiological Survey of Quality and Life at Work. Bivariate analyses and a multivariate logistic regression model (backward stepwise method) were employed to identify independent predictors, with model fitness assessed via the Akaike information criterion.

Results

Of the 210 residents, 151 (71.9%) reported a satisfactory QoWL. Multivariate analysis identified the following as significant protective factors: not smoking (odds ratio (OR) = 6.88, 95% confidence interval (CI) = 1.99-23.85), a work environment free of extreme temperatures (OR = 5.12, 95% CI = 1.49-17.63), adequate space distribution (OR = 4.17, 95% CI = 1.40-12.39), and the absence of work-related stress (OR = 7.34, 95% CI = 2.56-21.02).

Conclusions

This study demonstrates that specific environmental factors, primarily temperature control and spatial layout, alongside the mitigation of work-related stress, are the strongest predictors of a satisfactory QoWL. To foster a culture of prevention, hospital administrations should prioritize ergonomic workplace modifications and implement psychological support.

## Introduction

The Occupational Safety and Health Administration has described hospitals as workplaces with the highest risk to workers’ health, given that they have higher rates of occupational accidents and illnesses than almost any other line of work [1]. This unique environment within hospitals has led to studies on the quality of work life (QoWL) of healthcare personnel, particularly resident physicians [2-4].

Medical residency is a stage of professional development combining specialized training and clinical work under distinct conditions. On one hand, this work is considered a “special job,” where resident physicians carry out healthcare work in the hospital under particular working conditions. On the other hand, they are in a specialized professional training stage, fulfilling the obligations and responsibilities of postgraduate studies [5].

The International Organization for Standardization emphasizes the evaluation of these academic-work environments to safeguard human dignity [6] and ensure optimal health and safety standards, allowing for informed decision-making and resource allocation [7,8].

QoWL is a key component of resident physicians’ well-being that comprehensively assesses the relationship between working conditions, the fulfillment of their needs through their work activity, and the personal development attained [9-11].

Several models have been proposed for understanding QoWL achieved through the conditions of these academic-work environments. These frameworks emphasize that professional well-being arises from the synergy between individual needs and the organizational environment. According to these models, individual variables, such as health status and personal habits, and external factors, including the teaching environment, institutional regulations, organizational characteristics, and broader sociocultural factors, are not isolated elements. Instead, they function as integrated determinants of the resident physicians’ overall experience [10,12].

Particularly in tertiary care centers, where high clinical and academic complexity often converge to amplify stressors, understanding how these elements collectively influence the QoWL of residents is vital. Therefore, a new perspective that shifts the focus toward the proactive identification of specific determinants that foster a satisfactory QoWL is crucial. This study aimed to characterize and identify the organizational, environmental, and personal factors associated with a satisfactory QoWL among resident physicians.

## Materials and methods

Study design

We conducted a cross-sectional, analytical study within the Health Prevention and Promotion Service for Workers in a public tertiary care hospital in Mexico City that belongs to the social security system in Mexico. The project was submitted to and approved by the Ethics and Research Committees corresponding to the health institution (registration number: R-2022-785-020).

Sample and data collection

We utilized a convenience sampling method to recruit participants. We determined the required sample size using the Lwanga and Lemeshow formula for analytical research. The calculation was based on an expected measure of association with a predicted outcome proportion (P1) of 0.35 in the exposed group and 0.175 (P0) in the non-exposed group, assuming a precision level (E) of 0.3. This yielded a minimum requirement of 199 participants.

We included resident physicians from all medical specialties active during the study period, while those undertaking rotations or temporary stays were excluded to ensure the sample reflected the institution’s stable work environment. Resident physicians were invited to participate as they attended the institutional Health Prevention and Promotion Service for their standardized health and safety evaluations. This setting provided a controlled and private environment where the research objectives were explained, and written informed consent was obtained.

We collected data using web-based forms. The primary instrument was the CVT-GOHISALO, a Spanish scale validated on Mexican healthcare workers. It consists of 74 items, each scored on a five-point Likert scale. The instrument’s original validation study reported excellent internal consistency (Cronbach’s alpha = 0.911) and a robust seven-factor structure. Following the McCall T-score method, a raw score of 180 or higher (equivalent to a T-value ≥60) was established as the cutoff point for the primary outcome “satisfactory QoWL,” as specified in the instrument’s manual [9].

We collected information on possible associated factors, such as (1) sociodemographic data such as sex and age; (2) clinical characteristics of the individual, such as report of eating habits and physical and mental health status; (3) socio-environmental characteristics of the extra-laboral environment such as report of physical activity, consumption of tobacco, alcohol, and other substances, marital status and report of family aggression (specifically physical and/or verbal abuse); and finally (4) the characteristics of the academic-work environment such as seniority in the hospital, branch of medicine in which the resident works, work-related disabilities in the last year, as well as the perception of risk factors in the workplace (including physical, chemical, biological, ergonomic, and psychosocial factors) [10].

To assess these associated factors, we obtained an exhaustive clinical and occupational history for each participant, complemented by a battery of standardized, self-administered instruments: the Fagerström test for nicotine dependence, the Spanish version of the Alcohol Use Disorders Identification Test (AUDIT), and the IVAPT-PANDO inventory for assessing workplace violence [13-15]. The collected data were entered into a database, and two previously trained peer physicians reviewed the quality of the information.

Statistical analysis

Statistical analysis was performed using SPSS Statistics software, version 27.0 (IBM Corp., Armonk, NY, USA) [16]. To ensure data reliability, an initial quality assessment was performed to verify that no variables had missing data exceeding 10% of the sample. A descriptive analysis was performed to determine the academic and occupational characteristics of the physicians. The distribution of the quantitative variables was analyzed using skewness, kurtosis, and Gaussian methods, confirmed with the Kolmogorov-Smirnov test. These tests determined that age and years of service were freely distributed and are therefore presented as median and percentiles (p25-75). It should be noted that the objective of this study was to evaluate all possible factors that can lead to a satisfactory QoWL, so those measures of association >1 refer to those factors that favor the well-being of resident physicians.

For the bivariate analysis, Pearson’s chi-square test or Fisher’s exact test was used between each categorical factor as an independent variable and satisfactory QoWL as a dependent variable. For quantitative variables, the Mann-Whitney U test was used. Finally, for the multivariate model, multiple binary logistic regression analyses were used using the backward stepwise method, where all factors potentially associated in the literature that could predict the outcome of satisfactory QoWL were included. The model was chosen using clinical judgment in conjunction with the Akaike information criterion and the adjusted coefficient of determination. The p-values, odds ratios (ORs), and 95% confidence intervals (95% CIs) were analyzed, and a forest plot was also generated for the presentation of the multivariate analysis. Additionally, for the multivariate analysis, forest plots were generated using RevMan 5.4 to provide a standardized visual synthesis of the adjusted ORs and their corresponding 95% CIs. 

## Results

The study included 210 resident physicians, the majority of whom were men (133, 63.3%), with a median age of 29 years (28-31). Regarding their QoWL, 151 (71.9%) had a satisfactory level. The distribution of sociodemographic and clinical characteristics is presented in Table 1 and Table 2. While the overall study population consisted of 210 residents, slight variations in the totals reported for certain variables reflect missing data from participants who chose not to disclose that specific information.

**Table 1:** Clinical and demographic characteristics and homogeneity analysis between groups with satisfactory versus unsatisfactory QoWL among resident physicians. Quantitative variables are presented as median (P25–P75) due to their non-normal distribution. ^A^: U Mann-Whitney test; ^B^: Pearson’s chi-square test; ^C^: Fisher’s exact test; *: statistically significant. Note: Totals for some variables may not sum to the total due to missing data (non-response) in specific questionnaire items. QoWL = quality of work life

Baseline characteristics		Total (n = 210)	Unsatisfactory QoWL (n = 41)	Satisfactory QoWL (n = 151)	Test statistics	P-value
Age		29 (28-31)	29 (28-30)	29 (28-31)	2684^A^	0.186
Sex	Female	77 (36.7%	15 (36.6%)	56 (37.1%)	0.003^B^	0.953
	Male	133 (63.3%)	26 (63.4%)	95 (62.9%)		
Dietary habits report	Non adequate	149 (72.3%)	32 (78%)	104 (70.3%)	0.963^B^	0.327
	Adequate	57 (27.7%)	9 (22%)	44 (29.7%)		
Physical health status	Diagnosis of two or more physical comorbidities	23 (11.2%)	5 (12.2%)	16 (10.7%)	0.538^B^	0.764
	Diagnosis of one physical comorbidity	58 (28.2%)	10 (24.4%)	45 (30.2%)		
	No diagnosed physical comorbidities	125 (60.7%)	26 (63.4%)	88 (59.1%)		
Mental health status	Diagnosis of mental disorders	29 (14%)	5 (12.2%)	20 (13.4%)	1.000^C^	0.537
	No diagnosed mental disorders	178 (86%)	36 (87.8%)	129 (86.6%)		
Physical activity report	Sedentary/Light	64 (30.6%)	19 (46.3%)	40 (26.7%)	5.839^B^	0.016*
	Moderate/Intense	145 (69.4%)	22 (53.7%)	110 (73.3%)		
Tobacco consumption	Yes	33 (15.8%)	13 (31.7%)	16 (10.7%)	11.069^B^	<0.001*
	No	176 (84.2%)	28 (68.3%)	134 (89.3%)		
Alcohol consumption	Frequently	24 (11.4%)	7 (17.5%)	15 (10.1%)	2.378^B^	0.304
	Occasionally	128 (61%)	21 (52.5%)	95 (64.2%)		
	Never	53 (25.2%)	12 (30%)	38 (25.7%)		
Use of other substances	Yes	4 (1.9%)	1 (2.4%)	3 (2%)	0.030^B^	0.862
	No	205 (98.1%)	40 (97.6%)	147 (98%)		
Marital status	No partner at home	180 (87%)	35 (85.4%)	128 (86.5%)	0.034^B^	0.864
	With a partner at home	27 (13%)	6 (14.6%)	20 (13.5%)		
Report of domestic violence	Physical or verbal aggression	2 (1%)	1 (2.4%)	1 (0.7%)	0.976^B^	0.323
	None	207 (99%)	40 (97.6%)	(99.3%)		

**Table 2:** Learning and working environment characteristics and homogeneity analysis between groups with satisfactory versus unsatisfactory QoWL among resident physicians. Quantitative variables are presented as median (P25–P75) due to their non-normal distribution. ^A^: U Mann-Whitney test; ^B^: Pearson’s chi-square test; ^C^: Fisher’s exact test; ^*^: statistically significant. Note: Totals for some variables may not sum to the total due to missing data (non-response) in specific questionnaire items. QoWL = quality of work life

Learning and working environment characteristics		Total (n = 210)	Unsatisfactory QoWL (n = 41)	Satisfactory QoWL (n = 151)	Test statistics	P-alue
Residency years		2 (1-3)	2 (1-2)	2 (1-3)	2.503^A^	0.062
Medical service branch	Paraclinical area	17 (8.1%)	3 (7.3%)	14 (9.3%)	1.007^B^	0.909
	Inpatient care	171 (81.4%)	34 (82.9%)	119 (78.8%)		
	Surgical area	7 (3.3%)	1 (2.4%)	6 (4%)		
	Emergencies	7 (3.3%)	2 (4.9%)	5 (3.3%)		
	Intensive therapy	8 (3.8%)	1 (2.4%)	7 (4.6%)		
Work-related disabilities in the last year	Yes	7 (3.3%)	2 (4.9%)	4 (2.7%)	0.611^B^	0.384
	No	198 (96.3%)	39 (95.1%)	145 (97.3%)		
Perception of a learning and work environment free of	Noise	156 (76.5%)	26 (63.4%)	118 (79.7%)	4.711^B^	0.030*
	Vibrations	182 (89.2%)	35 (85.4%)	134 (90.5%)	0.909^B^	0.340
	Poor lighting in the working area	172 (84.3%)	30 (73.2%)	128 (86.5%)	4.152^B^	0.042*
	Extreme temperature	183 (89.7%)	31 (75.6%)	138 (93.2%)	10.550^B^	0.001*
	Radiation exposure	119 (57.8%)	17 (41.5%)	96 (64.4%)	7.036^B^	0.008*
	Dust from the preparation of medicines	173 (84.8%)	28 (68.3%)	132 (89.2%)	10.793^B^	0.001*
	Electrosurgical fumes	190 (93.1%)	35 (85.4%)	141 (95.3%)	4.917^B^	0.027*
	Anesthetic gases and vapors	181 (88.7%)	32 (78%)	134 (90.5%)	4.687^B^	0.030*
	Disinfectant liquids and solvents	174 (84.9%)	30 (73.2%)	129 (87.2%)	4.707^B^	0.030*
	Viruses, bacteria, and fungi	74 (35.6%)	14 (34.1%)	58 (37.3%)	0.141^B^	0.707
	Forced postures	130 (63.1%)	17 (41.5%)	105 (70%)	11.363^B^	<0.001*
	Physical overexertion	140 (68.6%)	19 (46.3%)	112 (75.7%)	12.988^B^	<0.001*
	Forced movements	174 (85.3%)	26 (63.4%)	136 (91.9%)	21.263^B^	<0.001*
	Stretching due to a lack of space	175 (85.8%)	28 (68.3%)	136 (91.9%)	15.578^B^	<0.001*
	Poor space layout	163 (79.9%)	24 (58.5%)	126 (85.1%)	13.870^B^	<0.001*
	Prolonged standing at work	88 (42.5%)	11 (26.8%)	72 (48%)	5.873^B^	0.015*
	Prolonged sitting at work	135 (65.9%)	22 (53.7%)	104 (69.3%)	3.524^B^	0.060
	Prolonged squatting at work	195 (95.6%)	38 (87.8%)	144 (97.3%)	6.379^B^	0.012*
	Workplace violence	186 (92.1%)	35 (85.4%)	137 (97.2%)	2.525^B^	0.112
	Work-related stress	146 (72.3%)	18 (45%)	117 (79.6%)	18.743^B^	<0.001*
	Sexual harassment	197 (98%)	39 (97.5%)	144 (98%)	0.032^B^	0.859

Table 1 shows the clinical and demographic characteristics and homogeneity analysis between groups with satisfactory versus unsatisfactory QoWL among resident physicians. In contrast, Table 2 describes the characteristics of the learning and work environment. When evaluating these factors, we found that the resident physicians mostly reported an adequate perception of the learning and work environment. It was observed that 197 (98%) medical residents reported an environment free of sexual harassment, and 186 (92.1%) reported that they did not suffer workplace violence. However, there was a high prevalence of certain occupational risk factors, with a frequency of 195 (95.6%) of prolonged squatting, 190 (83.1%) of exposure to electrosurgical fumes, and 183 (89.7%) to extreme temperature.

The bivariate analysis (Table 3) identified several factors associated with satisfactory QoWL, including not smoking (OR = 3.89, 95% CI = 1.68-8.99) and participating in moderate or intense physical activity (OR = 2.38, 95% CI = 1.17-4.84).

**Table 3:** Bivariate analysis between exposure factors and satisfactory QoWL in resident physicians. *: Statistically significant. All ORs >1 refer to factors that promote satisfactory QoWL. QoWL = quality of work life; OR = odds ratio; CI = confidence interval

Characteristics		Non-adjusted OR (95% CI)
Age		1.15 (0.97-1.37)
Sex	Female	Ref.
	Male	0.98 (0.48-2.0)
Dietary habits report	Non adequate	Ref.
	Adequate	1.50 (0.66-3.41)
Physical health status	Diagnosis of two or more physical comorbidities	Ref.
	Diagnosis of one physical comorbidity	1.06 (0.35-3.16)
	No diagnosed physical comorbidities	1.41 (0.417-4.74)
Mental health status	Diagnosis of mental disorders	Ref.
	No diagnosed mental disorders	0.90 (0.31-2.55)
Physical activity report	Sedentary/Light	Ref.
	Moderate/Intense	2.38 (1.17-4.84)*
Tobacco consumption	Yes	Ref.
	No	3.89 (1.68-8.99)*
Alcohol consumption	Frequently	Ref.
	Occasionally	1.48 (0.49-4.47)
	Never	2.11 (0.77-5.82)
Use of other substances	Yes	Ref.
	No	1.23 (0.12-12.10)
Marital status	No partner at home	Ref.
	With a partner at home	0.91 (0.34-2.44)
Report of domestic violence	Physical or verbal aggression	Ref.
	None	0.27 (0.02-4.39)
Residency years		1.26 (0.99-1.59)
Medical service branch	Paraclinical area	Ref.
	Inpatient care	0.75 (0.20-2.76)
	Surgical area	1.29 (0.11-15)
	Emergencies	0.54 (0.07-4.20)
	Intensive therapy	1.50 (0.13-17.18)
Work-related disabilities in the last year	Yes	Ref.
	No	1.86 (0.33-10.53)
Perception of a learning and work environment free of	Noise	2.27 (1.07-4.81)
	Vibrations	1.64 (0.59-4.58)
	Poor lighting in the working area	2.35 (1.02-5.42)*
	Extreme temperature	4.45 (1.71-11.62)*
	Radiation exposure	2.26 (1.26-5.18)*
	Dust from the preparation of medicines	0.26 (0.11-0.60)*
	Electrosurgical fumes	3.45 (1.09-10.92)*
	Anesthetic gases and vapors	2.69 (1.07-6.77)
	Disinfectant liquids and solvents	2.49 (1.07-5.78)*
	Viruses, bacteria, and fungi	1.15 (0.56-2.37)
	Forced postures	3.29 (1.62-6.72)*
	Physical overexertion	3.60 (1.75-7.40)*
	Forced movements	6.54 (2.75-15.57)*
	Stretching due to a lack of space	5.26 (2.17-12.73)*
	Poor space layout	4.06 (1.88-8.75)*
	Prolonged standing at work	2.52 (1.18-5.39)*
	Prolonged sitting at work	1.95 (0.97-3.92)
	Prolonged squatting at work	5.0 (1.28-19.57)*
	Workplace violence	2.35 (0.80-6.90)
	Work-related stress	4.77 (2.27-9.99)*
	Sexual harassment	1.23 (0.13-12.16)

Regarding the academic-work environment, a perception of workplace safety significantly promoted QoWL, specifically regarding the absence of radiation (OR = 2.26, 95% CI = 1.26-5.18), prolonged standing (OR = 2.52, 95% CI = 1.18-5.39), and physical overexertion (OR = 3.60, 95% CI = 1.75-7.40).

This positive association was also observed in environments free from work-related stress (OR = 4.77, 95% CI = 2.27-9.99), poor space layout (OR = 4.06, 95% CI = 1.88-8.75), and forced postures (OR = 3.29, 95% CI = 1.62-6.72). Finally, factors with wider CIs included forced movements (OR = 6.54, 95% CI = 2.75-15.57), lack of space (OR = 5.26, 95% CI = 2.17-12.73), and squatting (OR = 5.0, 95% CI = 1.28-19.57).

For the multivariate analysis, 28 models were obtained. The selection of the final multivariate model was guided by an integrative approach combining the Akaike Information Criterion, the marginal Cox and Snell R² (R²m = 0.29), and the adjusted R² (R²c = 0.411), with clinical judgment. This judgment was defined as the selection of variables based on their established biological and institutional plausibility. This ensured that the model prioritized predictors with high impact on occupational health, such as tobacco use and environmental stressors, regardless of their behavior in purely automated selection algorithms, thereby enhancing the model’s external validity and practical utility for hospital management.

**Figure 1:**
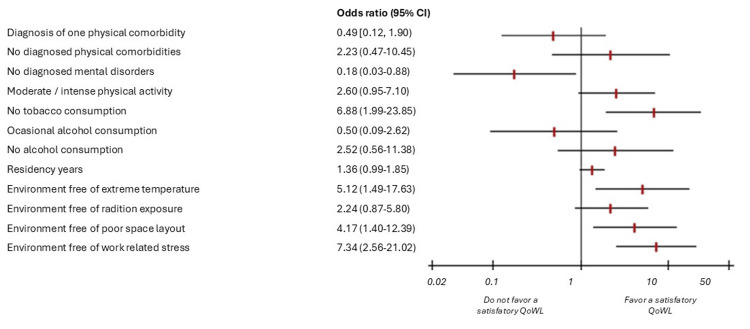
Multivariate analysis between exposure factors and satisfactory QoWL among medical residents. QoWL = quality of work life; OR = odds ratio; CI = confidence interval

The data from this model are presented in Table 4 and Figure 1, where the only variables that remained clinically relevant and statistically significant were not using tobacco (OR = 6.88, 95% CI = 1.99-23.85) for the socio-environmental characteristics. Among those related to the learning and work environment, we found that the perception of an environment without extreme temperatures (OR = 5.12, 95% CI = 1.49-17.63), free from poor distribution of space (OR = 4.17, 95% CI = 1.40-12.39), and without work-related stress (OR = 7.34, 95% CI = 2.56-21.02) were factors that favored a satisfactory QoWL. On the other hand, we observed that not having a personal history of mental health disorders was a risk factor (OR = 0.18, 95% CI = 0.03-0.88).

**Table 4:** Multivariate analysis between exposure factors and satisfactory QoWL in resident physicians. *: Statistically significant. All ORs >1 refer to factors that promote satisfactory QoWL. QoWL = quality of work life; OR = odds ratio; CI = confidence interval

Characteristics		Adjusted OR (95% CI)
Physical health status	Diagnosis of two or more physical comorbidities	Ref.
	Diagnosis of one physical comorbidity	0.49 (0.12-1.90)
	No diagnosed physical comorbidities	2.23 (0.47-10.45)
Mental health status	Diagnosis of mental disorders	Ref.
	No diagnosed mental disorders	0.18 (0.03-0.88)*
Physical activity report	Sedentary/Light	Ref.
	Moderate/Intense	2.60 (0.95-7.10)
Tobacco consumption	Yes	Ref.
	No	6.88 (1.99-23.85)*
Alcohol consumption	Frequently	Ref.
	Occasionally	0.50 (0.09-2.62)
	Never	2.52 (0.56-11.38)
Residency years		1.36 (0.99-1.85)
Perception of a learning and work environment free of	Extreme temperature	5.12 (1.49-17.63)*
	Radiation exposure	2.24 (0.87-5.80)
	Poor space layout	4.17 (1.40-12.39)*
	Work-related stress	7.34 (2.56-21.02)*

## Discussion

This study identified a broad range of factors that can optimize or affect satisfactory QoWL among resident physicians at a tertiary hospital in Mexico. A long-standing belief in medical residencies is that the resident physician is considered only an intern or student by the health institutions, contradicting reality, as residents are workers in the hospital centers, with a duality that gives them the academic status of a student, in addition to all the obligations, responsibilities, and labor rights of a health professional. In this way, it is urgent to conceptualize the “academic-labor environments” and elucidate all the factors to which they are exposed, which can favor or damage the QoWL of resident doctors [17]. The present study is strong because it considers the nature of the “special work” carried out by resident doctors and incorporates work and non-work elements that can impact QoWL [10,18].

This study observed that even though only a fifth of resident doctors had unsatisfactory QoWL levels, certain factors to which they were exposed can be reinforced or modified to improve them [19]. Specifically, organizational interventions aimed at stress reduction, ergonomic improvements in workplace layout, and the regulation of thermal comfort in clinical areas represent high-impact opportunities to enhance QoWL.

Regarding the learning and work environment, current recommendations limit the working week to 80 hours; however, looking at it another way, this represents almost half of the total hours. These long working days, which include night shifts, induce circadian misalignment and increase the risk of shift-work disorder among residents. From a physiological standpoint, this disruption of the circadian rhythm can generate work-related stress through the chronic exhaustion of energy reserves and the sustained activation of the hypothalamic-pituitary-adrenal axis, as evidenced by alterations in stress biomarkers [20].

In our study, a prevalence of work-related stress was observed in a third of resident doctors (36.7%); simultaneously, the perception of an environment free of work-related stress predicts QoWL. These results highlight the importance of monitoring the reduction of working hours to 80 per week, with minimum rest intervals between work periods and shifts. Nonetheless, a limitation of this analysis is the absence of a formal sleep quality evaluation. As sleep deprivation and poor hygiene are critical to resident well-being, future research should integrate objective sleep metrics to further elucidate the interplay between circadian health and QoWL.

Beyond these physiological and environmental factors, the psychological dimension also plays a crucial role in professional well-being. In contrast to what was shown in a previous study, not having a mental health diagnosis was a risk factor for a satisfactory QoWL (OR = 0.18, 95% CI = 0.03-0.88). While counterintuitive, this result aligns with the “healthy warrior effect” and the specialized coping strategies often observed in high-stress tertiary care environments. Residents with a documented history are frequently linked to professional support systems that provide them with therapeutic support and have established specialized coping strategies that their “apparently healthy” counterparts may lack [4].

Furthermore, those without a prior diagnosis may be more susceptible to the “stigma of vulnerability” in medical residency, leading to a denial of psychological distress and being more vulnerable to developing an unsatisfactory QoWL.

Our results emphasize the importance of including psychological support techniques within academic and work policies to provide the essential tools to cope with emotional challenges. This suggests that a satisfactory QoWL in tertiary care is not merely the absence of illness, but the result of proactive emotional and organizational support systems.

Regarding the work environment, the initial association between medicine dust exposure and QoWL likely acted as a proxy for clinical specialization and infrastructure quality. In tertiary care settings, medication preparation is concentrated in high-complexity units characterized by better resource allocation and specialized architectural layouts. Our multivariate analysis effectively decoupled these factors, demonstrating that optimal space distribution and thermal comfort, rather than chemical exposure, are the genuine environmental drivers of professional well-being.

For years, studies have evaluated how the physical environment can affect the health and satisfaction of workers. Regarding academic-work characteristics, environments free of extreme temperatures, whether cold or hot, are associated with a satisfactory QoWL. This is supported by the “unsafe behavior index,” postulated by Ramsey et al., where temperatures outside the 17°C to 23°C range are associated with diminished attention and concentration, leading to unsafe acts [21].

However, thermal comfort is only one component of a safe workspace. Regarding space layout, previous studies have coined the term “environmental quality,” which refers to the evaluation of functional comfort, that is, the degree to which the characteristics of the workspace help or hinder workers in their work [22]. Our results reinforce this knowledge, as in the multivariate analysis, it was observed that the perception of well-distributed spaces favors satisfactory QoWL, which is clinically relevant.

Although factors such as lighting are traditionally linked to workplace safety and error prevention, our model suggests that for residents in tertiary care, functional layout and thermal comfort are the main physical determinants of their professional well-being.

Regarding the socio-environmental characteristics of the extra-labor environment, not smoking is a factor that promotes a satisfactory QoWL, which reinforces the idea that in addition to the risks that smoking itself produces to health, the co-exposure to tobacco and some toxic substances found in work environments carries a greater risk of developing lung diseases, thus impacting the QoWL in its dimension of well-being achieved through work [23].

Furthermore, while our model identifies key environmental and psychological predictors and the variables included explain a significant percentage of the variance of the adjusted model (R²c = 0.405), it does not account for broader institutional factors. Elements such as hierarchical culture, feedback mechanisms, and psychosocial risk factors were beyond the scope of this assessment. Future research incorporating these institutional dimensions would provide a more comprehensive understanding of the systemic influences on residents’ QoWL [24].

Finally, as a single-site study conducted within a public tertiary hospital, the organizational culture and resource availability may not reflect other clinical settings, which restricts the generalizability of these findings. It is also important to note that the wide CIs observed for certain predictors indicate a degree of statistical imprecision likely linked to the sample size.

## Conclusions

Learning and working conditions and the set of individual and socio-environmental factors have a direct influence on QoWL and can facilitate or undermine its adjustment; therefore, it is important to consider the different elements that make up this academic-work environment, as they can alter a person’s model of QoWL, which comprises the well-being in their work environment and in their personal lives. To understand these dimensions, it is important to consider their contributions to the resident QoWL in an integrated manner. The characterization of factors, for example, stress management, thermal comfort, and space distribution, sets the basis for targeted interventions. Moreover, while personal habits, such as smoking, are an individual choice, they are also dependent on workplace health and safety policies. The current research highlights that the application of particular strategies, in conjunction with hospital-wide policies and an organizational culture of support, is fundamental for improving the quality of practice and academic-work environment for medical residents.
